# Patients Undergoing Myeloablative Chemotherapy and Hematopoietic Stem Cell Transplantation Exhibit Depleted Vitamin C Status in Association with Febrile Neutropenia

**DOI:** 10.3390/nu12061879

**Published:** 2020-06-24

**Authors:** Anitra C. Carr, Emma Spencer, Andrew Das, Natalie Meijer, Carolyn Lauren, Sean MacPherson, Stephen T. Chambers

**Affiliations:** 1 Nutrition in Medicine Research Group, Department of Pathology and Biomedical Science, University of Otago, Christchurch 8011, New Zealand; emma.spencer@otago.ac.nz; 2 Centre for Free Radical Research, Department of Pathology and Biomedical Science, University of Otago, Christchurch 8011, New Zealand; andrew.das@otago.ac.nz; 3 Department of Haematology, Christchurch Hospital, Christchurch 8011, New Zealand; natalie.meijer@cdhb.health.nz (N.M.); carolyn.lauren@cdhb.health.nz (C.L.); sean.macpherson@cdhb.health.nz (S.M.); 4 The Infection Group, Department of Pathology and Biomedical Science, University of Otago, Christchurch 8011, New Zealand; steve.chambers@otago.ac.nz

**Keywords:** vitamin C, ascorbate, ascorbic acid, immune compromised, conditioning chemotherapy, hematopoietic stem cell transplantation, inflammation, C-reactive protein, febrile neutropenia, oxidative stress

## Abstract

Patients undergoing myeloablative chemotherapy and hematopoietic stem cell transplantation (HSCT) experience profound neutropenia and vulnerability to infection. Previous research has indicated that patients with infections have depleted vitamin C status. In this study, we recruited 38 patients with hematopoietic cancer who were undergoing conditioning chemotherapy and HSCT. Blood samples were collected prior to transplantation, at one week, two weeks and four weeks following transplantation. Vitamin C status and biomarkers of inflammation (C-reactive protein) and oxidative stress (protein carbonyls and thiobarbituric acid reactive substances) were assessed in association with febrile neutropenia. The vitamin C status of the study participants decreased from 44 ± 7 µmol/L to 29 ± 5 µmol/L by week one (*p* = 0.001) and 19 ± 6 µmol/L by week two (*p* < 0.001), by which time all of the participants had undergone a febrile episode. By week four, vitamin C status had increased to 37 ± 10 µmol/L (*p* = 0.1). Pre-transplantation, the cohort comprised 19% with hypovitaminosis C (i.e., <23 µmol/L) and 8% with deficiency (i.e., <11 µmol/L). At week one, those with hypovitaminosis C had increased to 38%, and at week two, 72% had hypovitaminosis C, and 34% had outright deficiency. C-reactive protein concentrations increased from 3.5 ± 1.8 mg/L to 20 ± 11 mg/L at week one (*p* = 0.002), and 119 ± 25 mg/L at week two (*p* < 0.001), corresponding to the development of febrile neutropenia in the patients. By week four, these values had dropped to 17 ± 8 mg/L (*p* < 0.001). There was a significant inverse correlation between C-reactive protein concentrations and vitamin C status (*r* = −0.424, *p* < 0.001). Lipid oxidation (thiobarbituric acid reactive substances (TBARS)) increased significantly from 2.0 ± 0.3 µmol/L at baseline to 3.3 ± 0.6 µmol/L by week one (*p* < 0.001), and remained elevated at week two (*p* = 0.003), returning to baseline concentrations by week four (*p* = 0.3). Overall, the lowest mean vitamin C values (recorded at week two) corresponded with the highest mean C-reactive protein values and lowest mean neutrophil counts. Thus, depleted vitamin C status in the HSCT patients coincides with febrile neutropenia and elevated inflammation and oxidative stress.

## 1. Introduction

Hematopoietic stem cell transplantation (HSCT), using stem cells derived from bone marrow or peripheral blood, is reserved for patients with life-threatening diseases such as hematopoietic malignancies (e.g., leukemia, lymphoma, and myeloma). Disease free survival is low, with recurrence of underlying disease the main cause of death post-transplant [1]. HSCT has major treatment-related complications. Patients are typically treated with high-dose chemotherapy, with or without radiotherapy, to destroy the bone marrow’s ability to produce new blood cells, called myeloablative conditioning. Allogeneic transplant recipients also require immunosuppressive agents to prevent rejection of the donor stem cells. Infection is a common complication of both myeloablative conditioning and immunosuppressive agents.

Vitamin C is an essential nutrient with antioxidant and anti-inflammatory properties [2]. Since the middle of the last century, it has been known that patients with hematological cancers have significantly lower vitamin C status than healthy controls [3,4]; findings which have been confirmed in more recent case-control studies [5,6,7]. The reasons for these differences are uncertain, although elevated biomarkers of oxidative stress have been detected in patients with hematological cancer compared with healthy controls [6,7]. Myeloablative chemotherapy causes oxidative stress, inflammation, and tissue damage, which contribute to common side effects such as gastrointestinal mucositis [8,9,10]. Vitamin C concentrations have been shown to drop dramatically following conditioning chemotherapy [11,12,13]. These values continued to decline by week two and took at least one month to recover to pre-chemotherapy concentrations [12,13].

An explanation for the continued decrease in vitamin C status two weeks following myeloablative conditioning is acute infection developing in the presence of neutropenia (febrile neutropenia). Our previous research has indicated that depleted vitamin C status and enhanced biomarkers of oxidative stress (protein carbonyls) are frequently found in patients with severe infections [14,15]. Elevated oxidative stress may be both a cause and a consequence of the low vitamin C status of these patients. We also observed an association between depleted vitamin C status and elevated C-reactive protein, a marker of infection severity [16]. Similarly, Nannya and colleagues observed an inverse correlation between vitamin C and C-reactive protein in 15 patients undergoing allogeneic stem cell transplantation [12]. Thus, development of infection in the immune-compromised patients may contribute to further inflammation and oxidative stress and result in additional loss of vitamin C. Therefore, we tested this hypothesis in a cohort of HSCT recipients undergoing myeloablative conditioning by measuring vitamin C status in association with febrile neutropenia and markers of inflammation and oxidative stress.

## 2. Materials and Methods 

### 2.1. Study Participants 

Patients undergoing myeloablative conditioning and HSCT were recruited for this observational study (July 2017 to June 2018). All patients were cared for by the Hematology Department of Christchurch Hospital, a tertiary referral hospital servicing more than 600,000 people. Ethical approval was obtained from the New Zealand Southern Health and Disability Ethics Committee (#16STH235). All patients aged ≥18 years who were undergoing autologous or allogeneic transplants and able to provide signed informed consent were eligible. Patient data collected included the following: demographics (age, sex, ethnicity); hematological cancer diagnosis (multiple myeloma, Hodgkin and non-Hodgkin lymphoma, acute myeloid leukemia); transplant type (autologous or allogeneic); and conditioning chemotherapy regimen. Other data collected included the following: comorbidities; febrile episodes using Systemic Inflammatory Response Syndrome (SIRS) criteria (tachycardia (heart rate > 90 beats/min), tachypnea (respiratory rate > 20 breaths/min), and fever (temperature > 38 °C)); neutropenia (neutrophil counts < 1.5 × 10^9^/L); type and dose of antimicrobial agents used; blood culture information; and whether the patient was taking vitamin C-containing supplements or receiving enteral or parenteral nutrition, including type and dose. 

### 2.2. Blood Sampling and Processing

Blood samples were collected the day prior to transplant (baseline, week 0), seven days later (week 1), at day 10–14 (week 2), when the patients were exhibiting febrile neutropenia, and at approximately day 28 (week 4). The non-fasting blood samples (with heparin anticoagulant) were placed on ice and immediately transferred to the laboratory for centrifugation to separate plasma for vitamin C and oxidative stress biomarker analysis. An aliquot of the plasma was treated with an equal volume of 0.54 M perchloric acid and 100 µmol/L of the metal chelator diethylenetriamine-pentaacetic acid (DTPA) to precipitate protein and stabilize the vitamin C. The supernatant and spare plasma samples were stored at −80 °C until analysis.

### 2.3. Analysis of Blood Analytes

Routine hematological, kidney function, and liver function tests were carried out at Canterbury Health Laboratories, an International Accreditation New Zealand (IANZ) laboratory. C-reactive protein concentrations were assessed using endpoint nephelometry. The vitamin C content of the processed plasma samples was determined using HPLC with electrochemical detection, as described previously [17]. Systemic protein oxidation was determined by measuring plasma protein carbonyl content using a sensitive ELISA method, as described previously [18]. Plasma lipid oxidation was measured using the thiobarbituric acid reactive substances (TBARS) assay [19]. Fluorometric detection at 540 nm excitation and 590 nm emission provided greater sensitivity and specificity than spectrophotometric detection at 532 nm. Icteric samples were excluded from TBARS analyses due to interference [20]. 

### 2.4. Statistical Analysis

Data are presented as mean and *SD* or mean and 95% CI, as indicated. Statistical analyses were carried out using Microsoft Excel data analysis add-in (Microsoft, Auckland, NZ) and GraphPad Prism 8.0 software (Graphpad, San Diego, CA, USA). Differences between groups were determined using Student’s *t*-test or Mann–Whitney U test for non-parametric variables. Linear regression analyses were carried out using Pearson correlations. Statistical significance was set at *p* < 0.05. 

## 3. Results

### 3.1. Participant Characteristics

We recruited 38 patients undergoing conditioning chemotherapy and HSCT. The participant characteristics are indicated in Table 1 below. Of the total cohort, 88% identified as European and 12% as Māori. The predominant diagnoses were multiple myeloma and lymphoma, with a majority of the patients undergoing autologous transplant. Melphalan monotherapy was used for patients with multiple myeloma and carmustine combination therapy for patients with lymphoma.

### 3.2. Vitamin C Status of Study Participants

**Figure 1:**
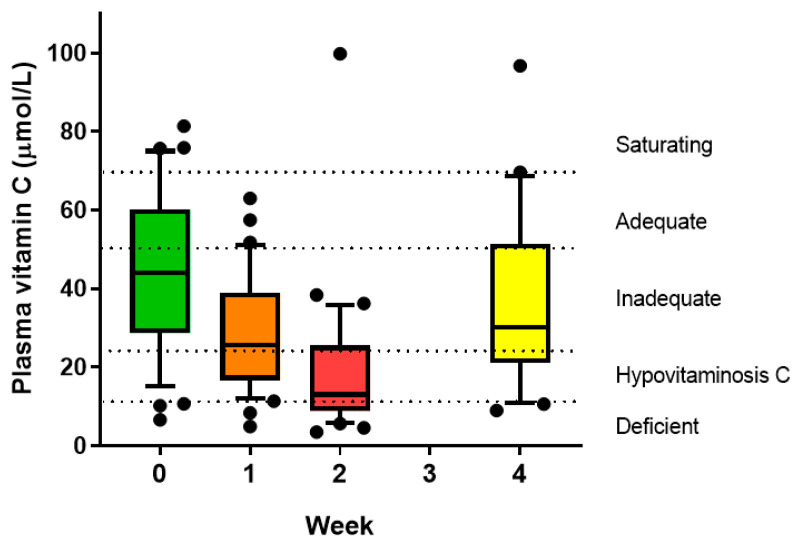
Vitamin C status of individuals undergoing myeloablative conditioning and hematopoietic stem cell transplantation (HSCT). Box plots show median with 25th and 75th percentiles as boundaries, and whiskers are the 10th and 90th percentiles, with symbols indicating outlying data points. Vitamin C category cut-offs are indicated by dotted lines. The mean values for weeks 1 and 2 were significantly lower than baseline (*p* ≤ 0.001).

The vitamin C status of the non-fasting participants pre-transplantation was 44 ± 7 µmol/L (Figure 1). This dropped to 29 ± 5 µmol/L one week following transplantation (*p* = 0.001); three of the participants had developed febrile neutropenia. Vitamin C concentrations dropped further to 19 ± 6 µmol/L another week later (*p* < 0.001), by which time all but one of the participants had developed febrile neutropenia. By week four, the vitamin C status of the participants had started to recover (38 ± 10 µmol/L) and was no longer significantly different to the pre-transplantation values (*p* = 0.1).

One of the participants (with multiple myeloma, treated with melphalan and autologous transplant) had unusually high vitamin C status (~100 µmol/L) at weeks two and four follow-up; the reason for this is unknown, as the patient did not report any vitamin C supplementation during this period. Of note, this was the only patient who did not experience an episode of febrile neutropenia (neutrophil counts at week two were 2.2 × 10^9^/L, and temperature and heart rate did not meet SIRS criteria).

**Figure 2:**
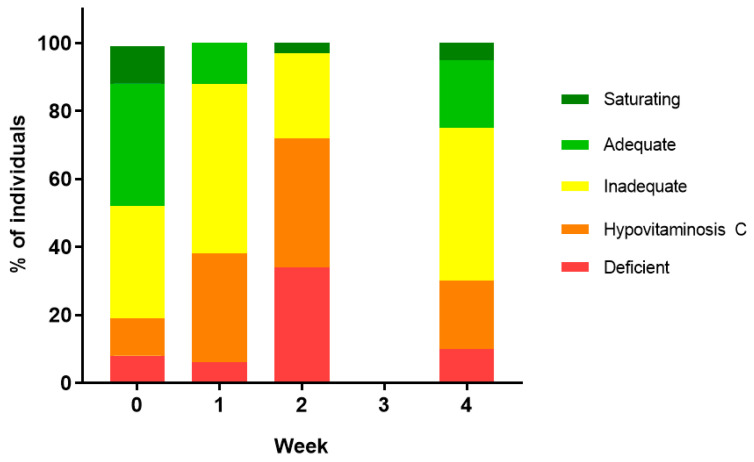
Percentage of individuals in different vitamin C categories. Vitamin C categories are presented as follows: saturating (>70 µmol/L), adequate (>50 µmol/L), inadequate (<50 µmol/L), hypovitaminosis C (<23 µmol/L), and deficient (<11 µmol/L).

Pre-transplantation, 53% of the patients had an inadequate vitamin C status (i.e., <50 µmol/L), 19% had hypovitaminosis C (i.e., <23 µmol/L), and 8% had vitamin C deficiency (i.e., <11 µmol/L) (Figure 2). One week post-transplantation, the number of patients with inadequate status had increased to 88% and those with hypovitaminosis C to 38%. Another week later, when all but one of the patients had developed febrile neutropenia, 72% had hypovitaminosis C and 34% had outright vitamin C deficiency. Although there was a decrease in these values by week four, to 75% with inadequate vitamin C status, 30% with hypovitaminosis C, and 10% with vitamin C deficiency, these were still higher than baseline values. 

### 3.3. C-Reactive Protein Concentrations Relative to Vitamin C Status

**Figure 3:**
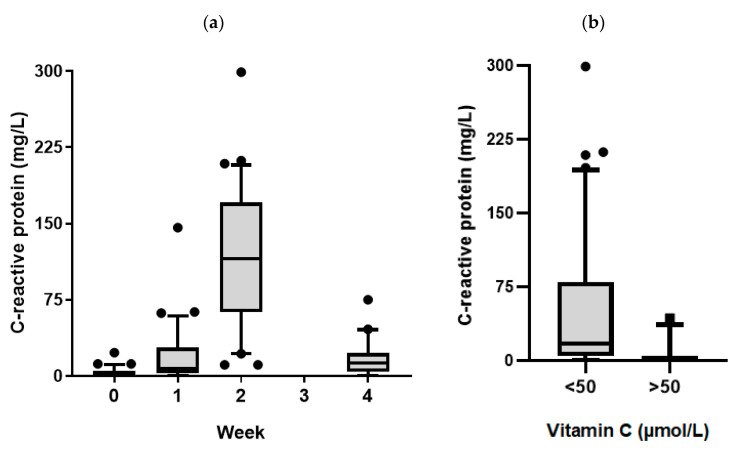
C-reactive protein concentrations in the patients. The C-reactive protein concentrations were assessed relative to (**a**) time of sampling, and (**b**) vitamin C concentration (< or >50 µmol/L, i.e., adequate). Box plots show median with 25th and 75th percentiles as boundaries, and whiskers are the 10th and 90th percentiles, with symbols indicating outlying data points. The mean values of weeks 1, 2, and 4 were significantly higher than baseline (*p* < 0.01); <50 µmol/L values were significantly higher than >50 µmol/L values (*p* < 0.001).

C-reactive protein mean plasma concentrations increased from 4 ± 2 mg/L (normal < 3 mg/L) at baseline to 20 ± 11 mg/L one week following transplantation (*p* = 0.002; Figure 3a). By week two, there was a large increase in mean C-reactive protein concentrations (119 ± 25 mg/L, *p* < 0.001), corresponding to development of febrile neutropenia in the patients (Table 2). By week four, these values had dropped to 17 ± 8 mg/L, although these were still significantly higher than baseline values (*p* < 0.001). There was an inverse correlation between C-reactive protein concentrations and vitamin C status, with higher vitamin C corresponding to lower C-reactive protein (*r* = −0.424, *p* < 0.001). At concentrations of vitamin C < 23 µmol/L (hypovitaminosis C), all but one patient had elevated C-reactive protein values (i.e., ≥3 mg/L); mean C-reactive protein was 71 ± 20 mg/L at <23 µmol/L vitamin C versus 21 ± 12 mg/L at >23 µmol/L vitamin C (*p* < 0.001). At concentrations of vitamin C < 50 µmol/L, mean C-reactive protein concentrations were 52 ± 14 mg/L versus 4 ± 4 mg/L at vitamin C concentrations > 50 µmol/L (*p* < 0.001; Figure 3b).

### 3.4. Oxidative Stress Biomarkers

Protein carbonyls were 172 ± 19 pmol/mg protein at baseline (week 0). These did not differ significantly over the time course (*p* > 0.05), and were not significantly different to a healthy cohort of comparable age (57 ± 17 years, *n* = 50) that we have previously measured (i.e., 159 ± 11 pmol/mg protein) [14]. In contrast, TBARS increased significantly from 2.0 ± 0.3 µmol/L at baseline to 3.3 ± 0.6 µmol/L by week one (*p* < 0.001), and remained elevated at week two (*p* = 0.003), returning to baseline concentrations by week four (*p* = 0.3). The elevated TBARS were comparable to values we have previously observed in critically ill patients [15]. There was a significant inverse correlation between vitamin C status and TBARS (*r* = −0.306, *p* < 0.001). 

## 4. Discussion

Our study showed profound depletion of plasma vitamin C concentrations in patients undergoing conditioning chemotherapy and HSCT. This reached a nadir at week two (19 µmol/L), with 72% developing hypovitaminosis C and 34% with outright deficiency. These values are directly comparable to those observed in critically ill patients in intensive care [16]. The vitamin C concentrations had not quite recovered to baseline values by week four. This confirms earlier reports that showed similar vitamin C profiles in smaller patient cohorts [11,12,13]. Despite recovery to near baseline concentrations by week four, these values are still considered inadequate (i.e., <50 µmol/L). Although the blood samples were non-fasting, low vitamin C status moderates potential fluctuations in plasma concentrations, as a result of recent dietary intake, due to uptake of the vitamin by depleted tissues. C-reactive protein concentrations increased dramatically by week two, in association with neutropenia and infection. We showed an inverse correlation between vitamin C status and C-reactive protein, with lower vitamin C status associated with higher C-reactive protein concentrations, an association also reported by Nannya and coworkers [12].

We have previously observed elevated protein carbonyls in patients with severe infections [14,15]. However, no increase in protein carbonyls was observed in the current study, despite patients exhibiting evidence of infection at week two. We have previously hypothesized that reactive oxygen species generated by activated neutrophils could contribute to the elevated protein carbonyls observed in patients with severe infections [15]. As such, the lack of an increase in protein carbonyls at week two in our current study may be a reflection of neutropenia in the patients. We did, however, observe elevated TBARS, a marker of lipid peroxidation, at weeks one and two. Others have reported similar findings, with elevated TBARS observed in patients versus healthy controls [6,7], and in patients following conditioning chemotherapy [8]. Administration of high-dose vitamin C to the patients was found to enhance the vitamin C status of the patients and decrease the elevated TBARS [8]. Whether this was through the vitamin’s antioxidant or enzyme cofactor functions is unknown.

Due to the important and varied functions of vitamin C in the body, and the significant depletion of vitamin C in the hematopoietic cancer patients, it is anticipated that supplementation of these patients with vitamin C to restore adequate concentrations would be of benefit to the patients [21]. Up to 80% of HSCT recipients experience gastrointestinal mucositis, which is an ablation-related injury of the mucosal lining of the gastrointestinal tract, including the mouth and throat [22]. Oral mucositis is a painful and debilitating inflammatory condition that can prevent eating and drinking. Oral mucositis is thought to be initiated by free radical damage that activates an inflammatory response [23], and preliminary findings indicate an inverse association between vitamin C status and the severity of mucositis [13]. Supplementation of allogeneic HSCT recipients with vitamin C at doses of 2000 mg/d resulted in saturating vitamin C status (i.e., >70 µmol/L) and was associated with an improvement in graft versus host symptoms of the mucous membranes [24]. High-dose vitamin C administration to oncology patients receiving chemotherapy has been shown to decrease toxicity to the gastrointestinal system [25], and to improve patient quality of life through decreasing nausea/vomiting and improving appetite [26]. This may be particularly important for patients who undergo repeated cycles of chemotherapy.

Cutting-edge research over the past decade has indicated that vitamin C has a role in epigenetic regulation through acting as a cofactor for enzymes that modify DNA and histones [27]. Cell culture and preclinical studies indicate that vitamin C regulates hematopoietic cell function and blocks leukemia progression through epigenetic mechanisms [28,29]. Recent research has indicated that supplementation of myeloid cancer patients with 500 mg/d vitamin C restored vitamin C concentrations to the normal range and was associated with epigenetic alterations in myeloid cells [30]. Preliminary evidence has also indicated that administration of high-dose vitamin C to patients with leukemia may improve mortality via epigenetic mechanisms [31,32]. Thus, vitamin C exhibits pleiotropic roles in hematological cancer therapy, decreasing chemotherapy-related toxicity and side effects, and potentially improving survival via epigenetic mechanisms.

## 5. Conclusions

Our study showed profound depletion of vitamin C in HSCT recipients. The nadir of vitamin C status corresponded with febrile neutropenia and elevated inflammation and oxidative stress. Clinical trials are currently underway to assess the potential effects of vitamin C administration in these patients.

## Figures and Tables

**Table 1:** Participant characteristics.

Characteristic	Total Cohort (*n* = 38)
Age, years ^1^	57 (8)
Male sex, *n* (%)	22 (58)
Diagnosis, *n* (%)	
Multiple myeloma	23 (61)
Lymphoma	12 (32)
Acute myeloid leukemia	3 (8)
Transplant, *n* (%)	
Autologous	32 (86)
Allogeneic	5 (14)
Conditioning regimen, *n* (%)	
Melphalan	23 (61)
Carmustine, Cytarabine, Etoposide, Melphalan	8 (22)
Carmustine, Thiotepa	1 (3)
Alemtuzumab, Fludarabine, Melphalan	1 (3)
Fludarabine, Cytarabine, Amsacrine, Busulfan, Anti-thymocyte globulin	1 (3)

^1^ Data is presented as mean (*SD*) unless specified otherwise.

**Table 2:** Vitamin C status relative to febrile neutropenia and oxidative stress.

Analyte	Week 0	Week 1	Week 2	Week 4
Vitamin C (µmol/L)	44 (7) ^1^	29 (5) *	19 (6) *	38 (10)
C-reactive protein (mg/L)	3.5 (1.8)	20 (11) *	119 (25) *	17 (8) *
Neutrophils (×10^9^/L)	3.2 (0.5)	1.8 (1.0) *	0.1 (0.1) *	2.1 (0.6) *
TBARS (µmol/L) ^2^	2.0 (0.3)	3.3 (0.6) *	2.9 (0.5) *	2.5 (0.6)

^1^ Data is presented as mean (95% CI); ^2^ TBARS, thiobarbituric acid reactive substances: one participant had icteric samples, so their data was excluded from TBARS analyses; * Significantly different to week 0 values (*p* < 0.01).
